# Differential Acute and Long Term Actions of Succinic Acid Monomethyl Ester Exposure on Insulin-Secreting BRIN-BD11 Cells

**DOI:** 10.1155/EDR.2001.19

**Published:** 2001

**Authors:** Sally F. Picton, Peter R. Flatt, Neville H. McClenaghan

**Affiliations:** 1 School of Biomedical Sciences University of Ulster Coleraine, N Ireland BT52 1SA UK

## Abstract

Esters of succinic acid are potent insulin secretagogues, and have been proposed as novel antidiabetic agents for type 2 diabetes. This study examines the effects of acute and chronic exposure to succinic acid monomethyl ester (SAM) on insulin secretion, glucose metabolism and pancreatic beta cell function using the BRIN-BD11 cell line. SAM stimulated insulin release in a dose-dependent manner at both non-stimulatory (1.1mM) and stimulatory (16.7mM) glucose. The depolarizing actions of arginine also stimulated a significant increase in SAM-induced insulin release but 2-ketoisocaproic acid (KIC) inhibited SAM induced insulin secretion indicating a possible competition between the preferential oxidative metabolism of these two agents. Prolonged (18hour) exposure to SAM revealed decreases in the insulin-secretory responses to glucose, KIC, glyceraldehyde and alanine. Furthermore, SAM diminished the effects of nonmetabolized secretagogues arginine and 3-isobutyl-1-methylxanthine (IBMX). While the ability of BRIN-BD11 cells to oxidise glucose was unaffected by SAM culture, glucose utilization was substantially reduced. Collectively, these data suggest that while SAM may enhance the secretory potential of non-metabolized secretagogues, it may also serve as a preferential metabolic fuel in preference to other important physiological nutrients and compromise pancreatic beta cell function following prolonged exposure.


*Int. Jnl. Experimental Diab. Res.*, Vol. 2, pp. 19–27
Reprints available directly from the publisher
Photocopying permitted by license only

© 2001 OPA (Overseas Publishers Association) N.V.
Published by license under
the Harwood Academic Publishers imprint,
part of Gordon and Breach Publishing,
a member of the Taylor & Francis Group.
Printed in the United States.

### Differential Acute and Long Term Actions of Succinic Acid Monomethyl Ester Exposure on Insulin-Secreting BRIN-BD11 Cells

SALLY F. PICTON, PETER R. FLATT and NEVILLE H. McCLENAGHAN*

*School of Biomedical Sciences, University of Ulster, Coleraine, N Ireland, BT52 1SA, UK*

*(Received 1 July 2000; In final form 1 October 2000)*

**Esters of succinic acid are potent insulin secretagogues, and have been proposed as novel antidiabetic agents for type 2 diabetes. This study examines the effects of acute and chronic exposure to succinic acid monomethyl ester (SAM) on insulin secretion, glucose metabolism and pancreatic beta cell function using the BRIN-BD11 cell line. SAM stimulated insulin release in a dose-dependent manner at both non-stimulatory (1.1mM) and stimulatory (16.7mM) glucose. The depolarizing actions of arginine also stimulated a significant increase in SAM-induced insulin release but 2-ketoisocaproic acid (KIC) inhibited SAM induced insulin secretion indicating a possible competition between the preferential oxidative metabolism of these two agents. Prolonged (18hour) exposure to SAM revealed decreases in the insulin-secretory responses to glucose, KIC, glyceraldehyde and alanine. Furthermore, SAM diminished the effects of non-metabolized secretagogues arginine and 3-isobutyl-1-methylxanthine (IBMX). While the ability of BRIN-BD11 cells to oxidise glucose was unaffected by SAM culture, glucose utilization was substantially reduced. Collectively, these data suggest that while SAM may enhance the secretory potential of non-metabolized secretagogues, it may also serve as a preferential metabolic fuel in preference to other important physiological nutrients and compromise pancreatic beta cell function following prolonged exposure.**

*Keywords*: Succinic acid monomethyl ester; Insulin secretion; Glucose metabolism; Clonal pancreatic beta cells

*Abbreviation*: IBMX, 3-isobutyl-1-methylxanthine; KIC, 2-ketoisocaproic acid; SAM, succinic acid monomethyl ester.

### INTRODUCTION

Succinic acid serves as an important cellular metabolic intermediate of glucose and mannose[1] and may act as a metabolic fuel through its rapid mitochondrial metabolism.[2] However, unlike glucose and other metabolizable nutrient fuels such as the amino acids,[3–5] succinic acid is not readily internalized into cells.[2,6] Addition of ester moieties to molecules has long served as an efficient means of internalizing compounds that are not otherwise transported across the plasma membrane.[7] Various esters of a diverse range of nutrients and nutrient metabolites have been demonstrated to exhibit enhanced biological and insulinotropic activity over their parent molecules.[8–14]

*Corresponding author. Tel.: +44-(0)28-7032-3011, Fax: +44-(0)28-7032-4965, e-mail: nh.mcclenaghan@ulst.ac.uk

Recent studies have demonstrated that various esters of succinic acid are rapidly internalized into pancreatic islets where they may evoke insulin secretion through increasing the supply of acetyl CoA and succinic acid to the Krebs cycle.[7,15-18] Esters of succinic acid, serving as potent insulin secretagogues, have therefore been proposed to be potential novel antidiabetic agents for the treatment of non-insulin-dependent diabetes mellitus.[6,8] However, to date, the apparent therapeutic potential of methyl esters of succinic acid has largely been based on their potent acute insulinotropic actions, with little attention focused on their long term effects on pancreatic beta cell function.

The present study examines both the acute and chronic effects of succinic acid monomethyl ester (SAM) on insulin secretion, glucose metabolism and pancreatic beta cell function using the clonal BRIN-BD11 cell line. The electrofusion-derived BRIN-BD11 cell line, represents a novel glucose-responsive insulin-secreting cell line with intact functional features of the parental pancreatic beta cell.[19-22] BRIN-BD11 cells have proven particularly useful for studying the effects of acute and prolonged exposure to a range of physiological and pharmacological agents, including amino acids and antidiabetic agents.[22-26] Through examining both the acute and chronic effects of SAM, the present investigation offers new insights into possible therapeutic application and the mechanisms by which this novel insulinotropic antidiabetic agent regulates pancreatic beta cell function.

### MATERIALS AND METHODS

### Chemicals

Reagents of analytical grade and deionized water (Purite, Oxon, UK) were used throughout. Unless otherwise stated, all chemicals were obtained from Sigma Chemical Company Ltd (Poole, Dorset, UK). RPMI 1640 tissue culture medium, foetal calf serum (FCS), antibiotics (100U/ml penicillin and 0.1mg/ml streptomycin), Hanks balanced salt solution (HBSS) and trypsin/EDTA (5.0g/l trypsin (1:250) and 2.0g/l EDTA in normal saline) were purchased from Gibco Life Technolgies Ltd (Paisley, Strathclyde, UK). Sodium dihydrogen orthophosphate and disodium hydrogen orthophosphate were obtained from BDH (UK). Rat insulin standard was purchased from Novo Industria (Copenhagen, Denmark), while $^{125}$I-bovine insulin, D-[5-$^{3}$H]glucose and D-[U-$^{14}$C]glucose were from Amersham International plc (Amersham, Bucks, UK). Optiphase HiSafe scintillation fluid was purchased from Wallac Scientific (UK).

### Culture of Clonal Insulin-Secreting Cells

BRIN-BD11 cells were routinely maintained in RPMI-1640 tissue culture medium supplemented with 11.1mM glucose, 10%(v/v) foetal calf serum, 100IU/ml penicillin and 0.1mg/ml streptomycin in a 37°C incubator with 5% $CO_2$ and 95% air, as described previously.[19] The BRIN-BD11 cells used for these studies were from passages 28-35.

### Acute Tests of Insulin Secretion

After seeding the cells at a density of $1.5 \times 10^5$ cells per well of 24-multiwell plates, BRIN-BD11 cells were left overnight at 37°C to attach as monolayers as described elsewhere.[19] BRIN-BD11 cells were then cultured for 18 hours in the presence or absence of 20mM SAM before performing acute tests of insulin release. Prior to testing, the RPMI-1640 culture medium was completely removed from each well and replaced with 1ml Krebs-Ringer bicarbonate (KRB) buffer, containing 115mM NaCl, 4.7mM KCl, 1.2mM $MgSO_4$, 1.28mM $CaCl_2$, 1.2mM $KH_2PO_4$, 25mM Hepes and 8.4%(w/v) $NaHCO_3$ (pH 7.4) supplemented with 0.5mg/ml bovine serum albumin and 1.1mM glucose. Monolayers of cells were then incubated for 40 minutes at 37°C, after which time the buffer was removed and replaced with 1ml KRB test buffer, supplemented with glucose, succinic acid monomethyl ester, and other test agents as detailed in the figures. After a 20min incubation (at 37°C), the test buffer was removed from each of the wells and stored at -20°C for subsequent measurement of insulin by radioimmunoassay as described previously.[27]

### Determination of Cell Viability and Cellular Insulin Content

Cell viability was determined by trypan blue exclusion.[19] For measurement of cellular insulin content, culture medium was removed from the monolayers (1.5 × $10^5$ cells per well) and 500µl acid-ethanol (1.5% (v/v) 0.7mol/l HCl, 75% (v/v) ethanol, 23.5% (v/v) $H_2O$) was added.[19] After an overnight incubation at 4°C, cells were disrupted and acid-ethanol extracts removed and stored at -20°C for determination of insulin.[27]

### Glucose Metabolism

Glucose oxidation and utilization were assessed after an 18h culture in the absence or presence of 20mM SAM using methods described previously.[28,29] Briefly, BRIN-BD11 cells were harvested, and groups of 2 × $10^5$ cells incubated at 37°C for 60 minutes at 1.1 or 16.7mM glucose in 40µl of KRB buffer containing either 1µCi of D-[U-$^{14}$C]glucose (glucose oxidation) or 1µCi D-[5-$^3$H]glucose (glucose utilization). In the case of glucose oxidation, the reaction was terminated after 60min by the addition of 50µl of 0.2M hydrochloric acid to the KRB buffer. Phenylethylamine (100µl) was then added to the filter paper placed on bottom of the vial for collection of $^{14}CO_2$ over a further 120 minute period (37°C). For glucose utilization experiments, the reaction was also terminated at 60min using 50µl 0.2M HCl, but in this case 500µl of $H_2O$ was added to the bottom of the vial to accumulate $^3H_2O$ aver a 15h period. Following addition of 5ml of aqueous (HiSafe) scinitillation fluid into the vial, $^3$H and $^{14}$C were measured using a beta counter. Counts obtained were corrected by means of a blank (processed as above but containing no cells) and a standard (containing only D-[U-$^{14}$C] glucose or D-[5-$^3$H] glucose).

### Statistical Analyses

Results are presented as mean ± SEM. Groups of data were compared in each case using unpaired Student's *t*-test and two-way analysis of variance in conjunction with Bonferroni's modified *t*-statistics. Differences were considered different if $p<0.05$.

### RESULTS

### Dose Responses to Succinic Acid Monomethyl Ester

Succinic acid monomethyl ester (SAM) stimulated 1.5- and 2.5-fold increases ($p<0.001$) in insulin secretion at 10 and 20mM respectively at non-stimulatory (1.1mM) glucose (Fig. 1). In the presence of a stimulatory (16.7mM) glucose, 10–20mM SAM similarly evoked a concentration-dependent 1.3-2.8-fold increase in insulin release ($p<0.001$) confirming its action as a potent insulin secretagogue in BRIN-BD11 cells (Fig. 1). Concentrations of SAM less than 10mM did not have an effect on insulin release (Fig. 1). Raising the glucose concentration from 1.1 to 16.7mM, evoked an increase in insulin release in the absence (2.2-fold, $p<0.001$) and presence (1.9–2.5 fold, $p<0.001$) of each concentration of SAM tested (Fig. 1).

### Effects of SAM and Insulinotropic Actions of Nutrients and 3-Isobutyl-1-Methylxanthine

As shown in Figure 2, 2-ketoisocaproic acid (KIC) and glyceraldehyde stimulated respective 3.4-fold and 2.1-fold increases ($p<0.001$) in insulin output at 16.7mM glucose, while the amino acids alanine and arginine evoked 5.5 fold and 2.6-fold increases ($p<0.001$), respectively. The phosphodiesterase inhibitor 3-isobutyl-1-methylxanthine (IBMX) also increased insuslin release 7.7-fold ($p<0.001$) from BRIN-BD11 cells. As shown in Figure 2, incubation with 20mM SAM increased insulin output in the presence of glyceraldehyde (1.3-fold increase, $p<0.05$) and arginine (1.9-fold increase, $p<0.001$). SAM

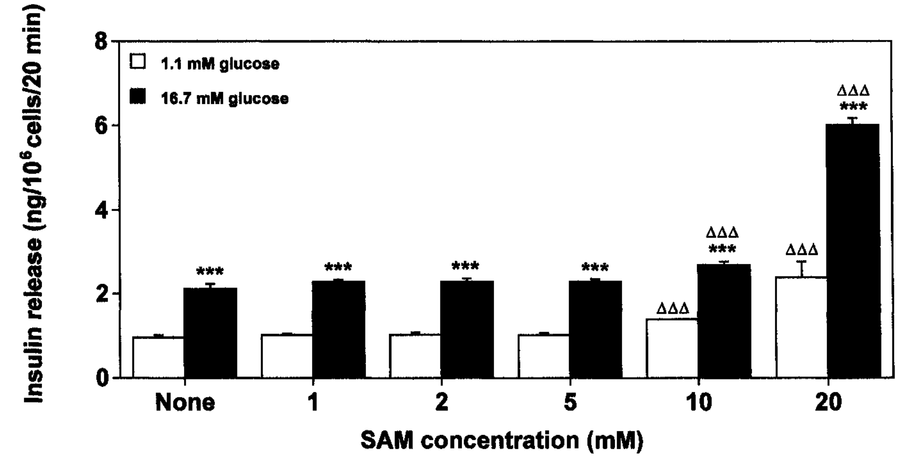

FIGURE 1 Effects of 0–20mM succinic acid monomethyl ester (SAM) on insulin secretion at non-stimulatory (1.1mM) and stimulatory (16.7mM) glucose. Following 40min of preincubation with a buffer containing 1.1mM glucose, effects of SAM were tested during a 20min incubation period. Values are mean ± SEM for 6 separate observations. ***$p<0.001$ compared with respective effects at 1.1mM glucose. $^{\Delta\Delta\Delta}p<0.001$ compared with effects in the absence of SAM.

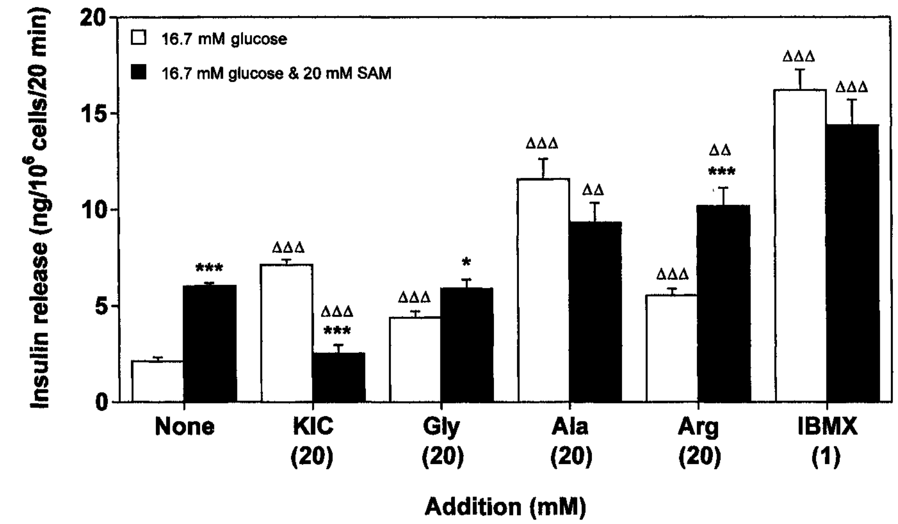

FIGURE 2 Effects of succinic acid monomethyl ester (SAM) on nutrient-induced insulin secretion. Following 40min of preincubation with a buffer containing 1.1mM glucose, effects of 20mM SAM in the absence or presence of 2-ketoisocaproic acid (KIC), D-glyceraldehyde (Gly), L-alanine (Ala), L-arginine (Arg) or 3-isobutyl-1-methylxanthine (IBMX) were tested during a 20min incubation period. Values are mean ± SEM for 6 separate observations. *$p<0.05$, ***$p<0.001$ compared with respective effects at 16.7mM glucose in the absence of SAM. $^{\Delta\Delta}p<0.01$, $^{\Delta\Delta\Delta}p<0.001$ compared with effects at 16.7mM glucose in the absence of addition.

(20mM) did not modify the stimulatory effects of either alanine or IBMX, but reduced insulin output induced by KIC by 65% ($p<0.001$) (Fig. 2). Interestingly, alanine ($p<0.01$), arginine ($p<0.01$) and IBMX ($p<0.001$) each increased insulin release over that of 16.7mM glucose and 20mM SAM alone, while glyceraldehyde exerted no influence and KIC decreased ($p<0.001$) insulin output (Fig. 2).

### Effects of Preincubation with SAM on Insulin Secretory Responsiveness

Preincubation of the monolayers with 20mM SAM caused a 1.4-fold increase in insulin release at 16.7mM glucose ($p<0.05$) (Fig. 3). However, preincubation with SAM resulted in a 52-54% decrease ($p<0.01$-$p<0.001$) in KIC- and glyceraldehyde-induced insulin output (Fig. 3). While, SAM preincubation exerted no influence on alanine-induced insulin secretion, it promoted a 2.3-fold increase ($p<0.001$) and a 35% decrease ($p<0.05$) in the effects of arginine and IBMX, respectively (Fig. 3). SAM preincubation increased subsequent insulin output (2.1 fold, $p<0.001$) at 1.1mM glucose (data not shown). While KIC and glyceraldehyde exerted no significant effect on SAM-induced insulin secretion at 16.7mM glucose following SAM preincubation, alanine, arginine and IBMX evoked 2.8-3.8-fold increases in insulin output (Fig. 3).

### Effects of Prolonged Exposure to SAM on BRIN-BD11 Cell Function

Prolonged (18hours) exposure to 20mM SAM effectively removed the ability of BRIN-BD11 cells to respond to stimulatory glucose or any other regulator tested (Fig. 4). SAM culture did not alter basal insulin release (at 1.1mM glucose) and insulin output in response to each secretagogue was not significantly greater than 1.1mM glucose alone (data not shown). While 18h culture with 20mM SAM exerted no significant effect on glucose oxidation (Fig. 5A), glucose utilization at both 1.1mM and 16.7mM glucose was significantly ($p<0.001$) decreased (Fig. 5B). It is important to note that culture with 20mM SAM neither decreased the insulin content (74.8 ± 3.2ng/$10^6$cells), nor the viability of the BRIN-BD11 cells, suggesting that the detrimental effects on metabolism and beta cell function were not simply due to a decreased cell number or reduced insulin content.

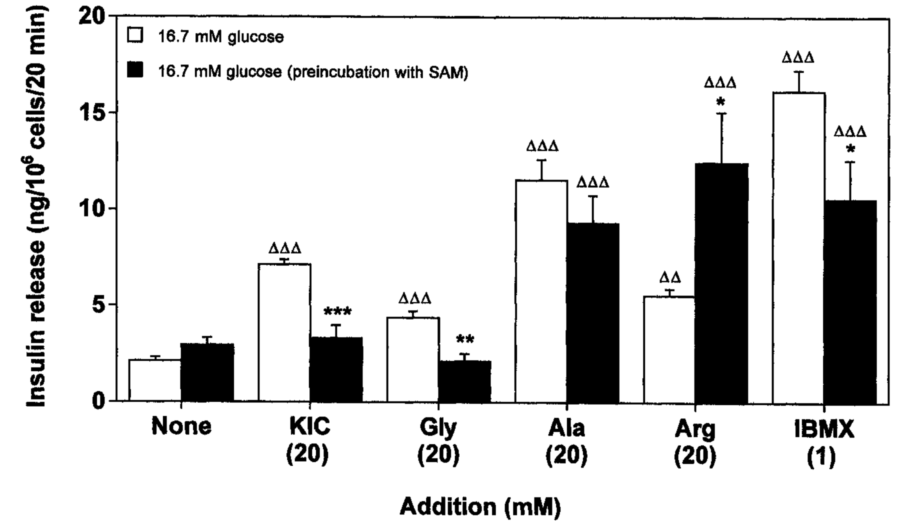

FIGURE 3 Effects of preincubation with succinic acid monomethyl ester (SAM) on nutrient-induced insulin secretion. Following 40min of preincubation with a buffer containing 1.1mM glucose in the absence or presence of 20mM SAM, effects of 2-ketoisocaproic acid (KIC), D-glyceraldehyde (Gly), L-alanine (Ala), L-arginine (Arg) or 3-isobutyl-1-methylxanthine (IBMX) were tested at 16.7mM glucose during a 20min incubation period. Values are mean ± SEM for 6 separate observations. *$p<0.05$, **$p<0.01$, ***$p<0.001$ compared with respective effects at 16.7mM glucose (preincubation without SAM). $^{\Delta\Delta}p<0.01$, $^{\Delta\Delta\Delta}p<0.001$ compared with effects in the absence of addition.

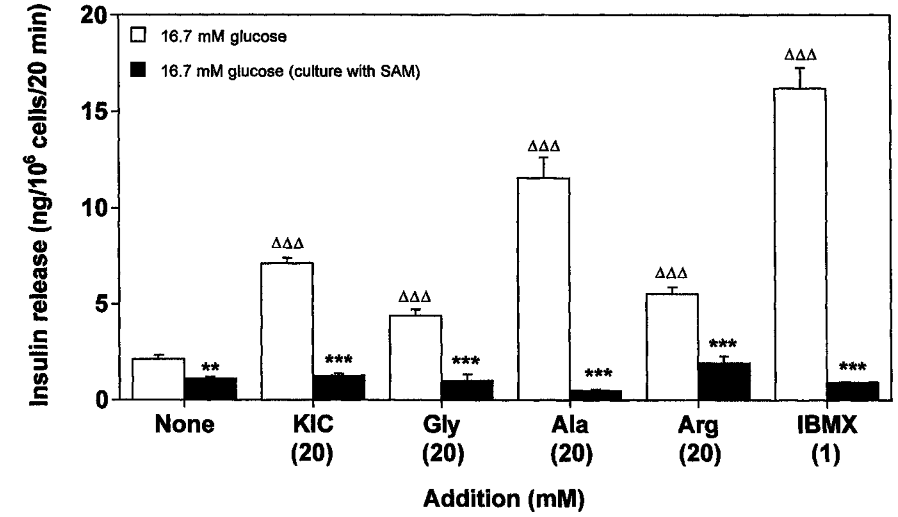

FIGURE 4 Effects of culture with succinic acid monomethyl ester (SAM) on nutrient-induced insulin secretion. After 18h culture in the absence or presence of 20mM SAM cells were preincubated for 40min with a buffer containing 1.1mM glucose and the effects of 2-ketoisocaproic acid (KIC), D-glyceraldehyde (Gly), L-alanine (Ala), L-arginine (Arg) or 3-isobutyl-1-methylxanthine (IBMX) were then tested at 16.7mM glucose during a 20min incubation period. Values are mean ± SEM for 6 separate observations. $^{**}p<0.01$, $^{***}p<0.001$ compared with respective effects at 16.7mM glucose (culture without SAM). $^{\Delta\Delta\Delta}p<0.001$ compared with effects in the absence of addition.

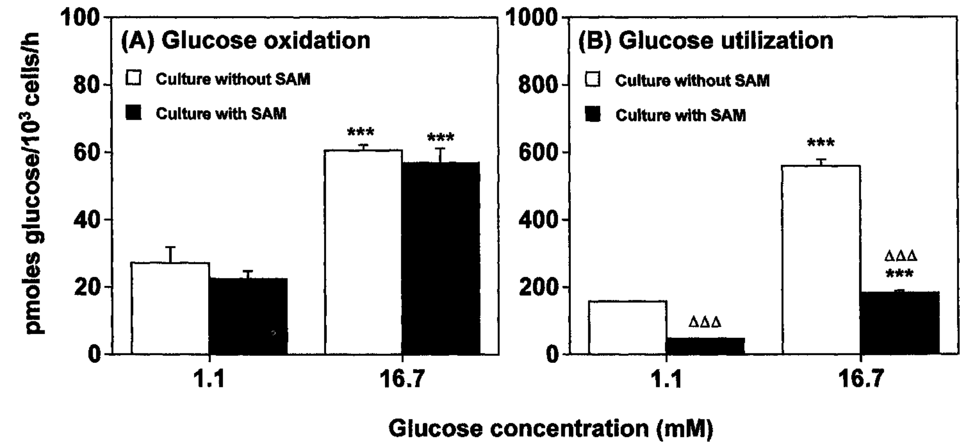

FIGURE 5 Effects of culture with succinic acid monomethyl ester (SAM) on (A) glucose oxidation and (B) glucose utilization. After 18h culture in the absence or presence of 20mM SAM, glucose oxidation and utilization were measured in the presence of 1.1 or 16.7mM glucose. Values are mean ± SEM for 4 separate observations. $^{***}p<0.001$ compared with respective effects at 1.1mM glucose. $^{\Delta\Delta\Delta}p<0.001$ compared with effects after culture without SAM.

### DISCUSSION

The present study examines the effects of acute and chronic exposure to SAM on insulin secretion, glucose metabolism and pancreatic beta cell function. Unlike its parent molecule, succinic acid, this ester is rapidly accumulated into pancreatic beta cells where it acts as an effective insulin secretagogue by virtue of its subsequent metabolism.[7,15] Consistent with previous studies on rat pancreatic islets,[7,15] acute exposure to SAM potently stimulated insulin secretion in a doserelated fashion from BRIN-BD11 cells at both non-stimulatory (1.1 mM) and stimulatory (16.7 mM) glucose concentrations. SAM was maximally effective both as an initiator and potentiator of insulin release at high concentrations (10–20 mM), as observed in normal islets.[7,15] The prominent increase in insulin secretory potency of SAM at a stimulatory as opposed to a non-stimulatory glucose concentration, indicate that SAM can utilize the metabolic and depolarizing actions of glucose in its insulinotropic effects. In addition, the present data also clearly indicate that BRIN-BD11 cells effectively transport SAM and utilize associated signal recognition pathways described in normal pancreatic beta cells.[7,15–18,30]

In addition to the characteristic glucose-responsiveness of BRIN-BD11 cells, notable insulin-secretory responses were also observed using a range of other important nutrient secretagogues and the phosphodiesterase inhibitor, IBMX.[30] As with glucose, SAM effectively utilized the stimulatory actions of the glycolytic fuel, glyceraldehyde to enhance its insulinotropic activity. Similarly, the depolarizing actions of arginine[21] were also able to stimulate a significant increase in SAM-induced insulin release. While, alanine and IBMX showed no synergistic actions with SAM, in the presence of KIC there was a significant inhibition of insulin release. KIC is a potent initiator of insulin secretion and is rapidly transported into beta cells[31,32] where it is readily available as a metabolic fuel.[33–35] The inhibitory effect of KIC on SAM-induced insulin release may reflect some form of competition in the oxidative metabolism of these two agents, which are immediately and completely utilized.

Consistent with the view that SAM may interfere with the metabolism of other secretagogues, prior exposure to SAM (during a 40 minute preincubation period) significantly decreased the subsequent responsiveness to KIC and glyceraldehyde. The secretory activity of IBMX was also reduced, and the insulinotropic potential of arginine, which does not serve as a significant fuel for beta cell metabolism[5,21] was significantly enhanced. Together these data suggest that SAM may, like glucose, enhance the secretory potential of non-metabolizable secretagogues,[21,36,37] and serve as a preferential metabolic fuel over that of other important physiological nutrients.

Prolonging the exposure of BRIN-BD11 cells to SAM to 18 hours had a detrimental effect on subsequent responsiveness to each of the agents tested. Indeed, in addition to abolishing secretory responses to glucose, KIC, glyceraldehyde and alanine, the effects of the potent non-metabolizable secretagogues arginine and IBMX were also curtailed. These effects were not attributable to a depletion of cellular insulin content or to a reduction in BRIN-BD11 cell viability, neither of which were affected by the culture conditions. In addition to altering secretory function, SAM-induced changes in the cellular metabolism of glucose, the principal physiological regulator of beta cell function.[30] While the ability of the cells to oxidise glucose was unaffected by chronic 18 h exposure to SAM, glucose utilization was substantially reduced in response to SAM culture. This observation suggests that cells retain the ability to metabolise glucose but after prior exposure to SAM, BRIN-BD11 cells are less likely to utilize glucose than before, perhaps as a result of establishing a preference for SAM. The possibility of a different anaplerotic function of succinic acid and KIC for the regulation of mitochondrial metabolism in these cells remains to be established.

Collectively these data reveal that SAM exerts differential actions on the regulation of pancreatic beta cell function depending on the nature and duration of exposure. The present study highlights the fact that SAM may modulate the insulinotropic actions of a number of agents acting at different sites in the pancreatic beta cell, suggesting the interaction or possible common signalling pathways used by SAM and each of the agents tested. In considering SAM as a potential antidiabetic agent for non-insulin-dependent diabetes, attention should be focussed on possible deleterious actions and potential reversibility of effects of long-term exposure to SAM on pancreatic beta cell function. While previous observations indicate that short-term exposure to SAM (120min) did not affect either glucose oxidation or glucose utilization,[7,15] the present *in vitro* study suggests that more prolonged exposure may substantially alter insulin-secretory responsiveness. These data merit substantiation by *in vivo* studies, but similar desensitization of insulin-secretory function has been observed with sulphonylureas both *in vivo* and *in vitro* during extended culture.[25,26,38,39] These findings clearly show the need for further research bearing in mind that SAM represents only one of an growing number of insulinotropic nutrient esters[8,10–14] which may prove to be better candidates for the future therapy of type 2 diabetes.

### *Acknowledgements*

These studies were supported in part by the Department of Health and Personal Social Services, Northern Ireland and University of Ulster Research Strategy funding.

### *References*

[1] Grodsky, G. M., Batts, A. A., Bennett, L. L., McWilliams, N. B. and Smith, D. F. (1963). Effects of carbohydrates on secretion of insulin form isolated rat pancreas. *Am. J. Physiol*, **205**, 638–644.
[2] McDonald, M. J. and Fahien, L. A. (1990). Insulin release in pancreatic islets by a glycolytic and Krebs cycle intermediate: contrasting patterns of glyceraldehyde and succinate. *Arch. Biochem. Biophys.*, **279**, 104–108.
[3] Guidotti, G. G., Borghetti, A. F. and Gazzola, G. C. (1978). The regulation of amino acid transport in animal cells. *Biochim. Biophys. Acta.*, **515**, 329–366.
[4] Collinari, E. J. and Oxender, D. L. (1987). Mechanisms of transport of amino acids across membranes. *Ann. Rev. Nutr.*, **7**, 75–90.
[5] McGivan, J. D. and Pastor-Anglada, M. (1994). Regulatory and molecular aspects of mammalian amino acid transport. *Biochem. J.*, **299**, 321–334.
[6] McDonald, M. J. and Fahien, L. A. (1988). Glyceraldehyde phosphate and methyl esters of succinic acid. Two "new" potent insulin secretagogues. *Diabetes*, **37**, 997–999.
[7] Malaisse, W. J. Rasschaert, J., Villanueva-Penacarrillo, M. L. and Valverde, I. (1993). Respiratory, ionic, functional effects of succinate esters in pancreatic islets. *Am. J. Physiol.*, **264**, E428–E433.
[8] Malaisse, W. J. (1995). The esters of carboxylic nutrients as insulinotropic tools in NIDDM. *Gen. Pharmacol.*, **26**, 1133–1141.
[9] Laghmich, A., Ladriere, L., Malaisse-Lagae, F., Dannacher, H., Bjorkling, F. and Malaisse, W. J. (1998). Stimulation of biosynthetic activity by novel succinate esters in rat pancreatic islets. *Biochem. Pharmacol.*, **55**, 909–913.
[10] Louchami, K., Kadiata, M. M., Jijakli, H. and Malaisse, W. J. (1998). Effects of the polyacetate esters of nutrient and nonnutrient monosaccharides on $^{45}$calcium efflux and release from perifused rat pancreatic islets. *Int. J. Pancreatol*, **24**, 103–109.
[11] Valverde, I., Vicent, D., Villanueva-Penacarrillo, M. L., Malaisse-Lagae, F. and Malaisse, W. J. (1997). Stimulation of insulin release *in vivo* by the methyl esters of succinic acid and glutamic acid. *Adv. Exp. Med. Biol.*, **426**, 231–234.
[12] Malaisse, W. J. (1999). Insulinotropic action of monosaccharide esters: therapeutic perspectives. *Diabetologia*, **42**, 286–291.
[13] Mercan, D., Kadiata, M. M. and Malaisse, W. J. (1999). Differences in the time course of the metabolic response of B and non-B pancreatic islet cells to D-glucose metabolized or non-metabolized hexose esters. *Biochem. Biophys. Res. Commun.*, **262**, 346–349.
[14] Laghmich, A. Ladriere, L., Dannacher, H., Bjorkling, F. and Malaisse, W. J. (2000). New esters of succinic acid and mixed molecules formed by such esters and a meglitinide analog: study of their insulinotropic potential. *Pharmacol Res.*, **41**, 543–554.
[15] Malaisse, W. J. and Sener, A. (1993). Metabolic effects and fate of succinic esters in pancreatic islets. *Am. J. Physiol.*, **264**, E434–E440.
[16] Vicent, D., Villanueva-Pencarrillo, M. L., Malaisse-Lagae, F., Leclerq-Meyer, V., Valverde, I. and Malaisse, W. J. (1994). *In vivo* stimulation of insulin release by succinic acid monomethyl esters. *Arch. Int. Pharm. Ther.*, **327**, 246–250.
[17] Ladriere, L., Laghmich, A., Malaisse-Lagae, F., Dannacher, H., Bjorkling, F. and Malaisse, W. J. (1997). Comparison between the insulinotropic potential of ten new esters of succinic acid. *European J. Pharmacol.*, **334**, 87–93.
[18] Ladriere, L., Louchami, K., Vinambres, C., Kadiata, M. M., Jijakli, H., Villanueva-Pencarrillo, M. L., Valverde, I. and Malaisse, W. J. (1998). Insulinotropic action of the monoethyl ester of succinic acid. *Gen. Pharmacol.*, **31**, 337–383.
[19] McClenaghan, N. H., Barnett, C. R., Ah-Sing, E., Abdel-Wahab, Y. H. A., O'Harte, F. P. M., Yoon, T.-W., Swanston-Flatt, S. K. and Flatt, P. R. (1996). Characterisation of a novel glucose responsive insulin-secreting cell line, BRIN-BD11, produced by electrofusion. *Diabetes*, **45**, 1132–1140.
[20] McClenaghan, N. H., Gray, A. M., Barnett, C. R. and Flatt P. R. (1996). Hexose recognition by insulin-secreting BRIN-BD11 cells. *Biochem. Biophys. Res. Comm.*, **223**, 724–728.
[21] McClenaghan, N. H., Barnett, C. R., O'Harte, F. P. M. and Flatt, P. R. (1996). Mechanisms of amino acid induced insulin secretion from the glucose-responsive BRIN-BD11 pancreatic B-cell line. *J. Endocrinol.*, **151**, 349–357.

[22] McClenaghan, N. H., Elsner, M., Tiedge, M. and Lenzen, S. (1998). Molecular characterization of the glucose-sensing mechanism in the clonal insulin secreting BRIN-BD11 cell line. *Biochem. Biophys. Res. Comm.*, **242**, 262–266.
[23] Rasschaert, J., Flatt, P. R., Barnett, C. R., McClenaghan, N. H. and Malaisse, W. J. (1996). D-Glucose metabolism in BRIN-BD11 islet cells. *Biochem. Mol. Med.*, **57**, 97–105.
[24] McClenaghan, N. H., Flatt, P. R. and Bailey, C. J. (1998). Insulin-releasing action of the novel antidiabetic agent BTS 67 582. *Br. J. Pharmacol.*, **123**, 400–404.
[25] Ball, A. J., McCluskey, J. T., Flatt, P. R. and McClenaghan, N. H. (2000). Drug-induced desensitization of insulinotropic actions of sulfonylureas. *Biochem. Biophys. Res. Comm.*, **271**, 234–239.
[26] McClenaghan, N. H., Ball, A. J. and Flatt, P. R. (2000). Induced desensitization of the insulinotropic effects of antidiabetic drugs, BTS 67 582 and tolbutamide. *Br. J. Pharmacol.*, **130**, 478–484.
[27] Flatt, P. R. and Bailey, C. J. (1981). Abnormal plasma glucose and insulin responses in heterozygous (ob/+) mice. *Diabetologia*, **20**, 573–577.
[28] Hellman, B., Sehlin, J. and Taljedal I. B. (1971). Effects of glucose and other modifiers of insulin release on the oxidative metabolism of amino acids micro-dissected pancreatic islets. *Biochem. J.*, **123**, 513–521.
[29] Malaisse-Lagae, F. and Malaisse, W. J. (1984). Insulin release by pancreatic islets. In: *Methods in Diabetes Research*, **1**, Larner, J. and Pohl, S. L. Eds., 147–152, Wiley, New York.
[30] Flatt, P. R. and Lenzen, S. (Eds.) (1994). *Frontiers of Insulin Secretion and Pancreatic B-Cell Research*. Smith-Gordon, London.
[31] Best, L., Treibilcock, R. and Tomlinson, S. (1992). 2-Ketoisocaproate transport in insulin-secreting cells. *Biosci. Rep.*, **12**, 69–76.
[32] McClenaghan, N. H. and Flatt, P. R. (2000). Metabolic and $K_{ATP}$ channel-independent actions of keto acid initiators of insulin secretion. *Pancreas*, **20**, 38–46.
[33] Lenzen, S. (1978). Effects of alpha-ketocarboxylic acids and 4-pentenoic acid on insulin secretion from the perfused rat pancreas. *Biochem. Pharmacol.*, **27**, 1321–1324.
[34] Panten, U., Holze, S. and Lenzen, S. (1980). Changes of function and metabolism of the pancreatic B-cell caused by amino acids and related compounds. *Horm. Metab. Res.*, **10**, 27–30.
[35] Lenzen, S. Formanek, H. and Panten, U. (1982). Signal function of metabolism of neutral amino acids and 2-keto acids for initiation of insulin secretion. *J. Biol. Chem:*, **257**, 6631–6633.
[36] McClenaghan, N. H., Barnett, C. R. and Flatt, P. R. (1997). Mechanisms of action of cationic amino acids on insulin secreting BRIN-BD11 cells. *Biochem. Soc. Trans.*, **25**, 144S.
[37] McClenaghan, N. H., Barnett, C. R. and Flatt, P. R. (1998). $Na^+$ cotransport by metabolizable and non-metabolizable amino acids stimulates a glucose-regulated insulin-secretory response. *Biochem. Biophys. Res. Commun.*, **249**, 299–303.
[38] Filiponni, P., Marcelli, M., Nicoletti, I., Pacifici, R., Saneusanio, F. and Brunetti P. (1983). Suppressive effect of long-term sulphonylurea treatment on A, B and D cells of normal rat pancreas. *Endocrinology*, **113**, 1972–1979.
[39] Karam, J. H., Sanz, N., Salamon, E. and Nolte, M. S. (1986). Selective unresponsiveness of pancreatic beta cells to sulfonylurea stimulation during sulfonylurea therapy in NIDDM. *Diabetes*, **35**, 1314–1320.

